# Conditional cash transfer programme: Impact on homicide rates and hospitalisations from violence in Brazil

**DOI:** 10.1371/journal.pone.0208925

**Published:** 2018-12-31

**Authors:** Daiane Borges Machado, Laura C. Rodrigues, Davide Rasella, Maurício Lima Barreto, Ricardo Araya

**Affiliations:** 1 Center of Data and Knowledge Integration for Health (CIDACS), Salvador, Bahia, Brazil; 2 Centre for Global Mental Health (LSHTM), London School of Hygiene & Tropical Medicine, London, United Kingdom; 3 London School of Hygiene & Tropical Medicine, London, United Kingdom; 4 King’s College London, London, United Kingdom; London School of Economics, UNITED KINGDOM

## Abstract

**Background:**

Homicide kills more people than war globally and is associated with income inequality. In Brazil, one of the most unequal countries of the world, the homicide rate is four times higher than the world average. Establishing if the Brazilian conditional cash transfer programme [Bolsa Familia Programme (BFP)], the largest in the world, is associated with a reduction in the rate of homicide is relevant for violence prevention programs. We aimed to assess the effect of BFP coverage on homicide and hospitalization rates from violence.

**Methods:**

BFP coverage and rates of homicide (overall and disaggregated by sex and age) and hospitalizations from violence from all 5,507 Brazilian municipalities between 2004 and 2012 were explored using multivariable negative binomial regression models with fixed effect for panel data. Robustness of results was explored using sensitivity analyses such as difference-in-difference models.

**Findings:**

Homicide rates and hospitalization from violence decreased as BFP coverage in the target population increased. For each percent increase in the uptake of the BFP, the homicide rate decreased by 0.3% (Rate Ratio:0.997; 95%CI:0.996–0.997) and hospitalizations from violence by 0.4% (RR: 0.996;95%CI:0.995–0.996). Rates of homicide and hospitalizations from violence were also negatively associated with the duration of BFP coverage. When, coverage of the target population was at least 70% for one-year, hospitalizations from violence decreased by 8%; two-years 14%, three-years 20%, and four years 25%.

**Interpretation:**

Our results support the hypothesis that conditional cash transfer programs might have as an additional benefit the prevention of homicides and hospitalizations from violence. Social protection interventions could contribute to decrease levels of violence in low-and-middle-income-countries through reducing poverty and/or socioeconomic inequalities.

## Introduction

Homicide is the most serious outcome of interpersonal violence, killing more people worldwide than all wars combined since 2000 [1]. Globally the homicide rate is 6.7 per 100 000 inhabitants per year; in low-middle-income American countries is 28.5 per 100 000, the highest rate worldwide [1]. From 2000 to 2012, homicides decreased by 39% in high-income countries, 13% in upper and lower middle-income countries, and 10% in low-income countries [1].

The homicide rate in Brazil is four times higher than the world average (26.2 per 100 000) [2]. It steadily increased since 1980 [3] but the overall rate stagnated between 2004–2014, with about 60 000 homicides per year [4]. Homicides and road traffic accidents are the main causes of shorter life expectancy for men in Brazil [5]. The groups at highest risk of homicide are men, young, black, and less educated [2,6]. For instance, homicide rates in Brazil are 12 times higher in men [2].

The situation is diverse inside Brazil: in the state of Rio de Janeiro and Sao Paulo homicide rates decreased by almost half; but in other states, like Rio Grande do Norte e Maranhão, they increased four times from 2004 to 2014 [7].

Homicide rates in Brazil are higher in areas of greater income inequality [8,6,2]. Brazil has changed economically in the last decade but large social inequalities and regional differences remain. The wealthier South and Southeast regions show better socio-economic indicators than the North and Northeast [9] were the homicide rates increased, mostly from 2004 to 2014 [7]. Around 10% of the Brazilian population lived below the poverty line [10] and 10% were illiterate [11] in 2010.

The Brazilian government introduced a conditional cash transfer programme, “Bolsa Família programme” (BFP), in 2003 [12] to promote social inclusion and strengthen human capital among the poorest. BFP removed 22.2 million Brazilians out of poverty; over 14 million families were receiving the benefits of BFP by 2014 [13]. To be eligible for BFP, the family monthly per-capita income should be <22 dollars or <44 dollars if the family includes a child, adolescent, or pregnant woman [13]. The programme uses conditionalities to promote behavioral change, such as children have to attend a minimum of 85% schooling days and women and children have to attend health care appointments as per requirements included in the programme. It is implied that making the benefits conditional upon ‘positive’ behaviors can further increase the chances of breaking out of the cycle of poverty through increased education or improved health.

There is some evidence that BFP can reduce poverty [14], inequalities [14], crime [15], child mortality [16], hospital admissions [16], and leprosy incidence [17] as well as increase school attendance [18].

The potential effect of BFP on homicide rates is uncertain. Studies of the effect of BFP on crime, showed conflicting results [18, 19, 20]. There are good reasons to believe that BFP might decrease the chance of an individual committing homicide or being a victim of a homicide. The programme aims to provide additional income to alleviate extreme poverty and reduce income inequalities, which are associated with homicide [21]. BFP can also help preventing homicides through increasing educational level, job opportunities, and income generation among the poorest families [10]. We aimed to investigate the potential effect of the BFP on homicide rates and on hospitalizations from violence in Brazil.

## Methods

Mixed ecological design using panel data from all the 5,507 Brazilian municipalities between 2004 and 2012.

### Data sources

Mortality data was collected from the Brazilian Ministry of Health’s Mortality Information System and hospitalization data from the Hospitalization Information System, both available at DATASUS [4]. Socioeconomic and demographic variables were obtained from the Brazilian Institute of Geography and Statistics [22]. BFP coverage was obtained from the Ministry of Social Development database [23].

All these three data sources are fully online available and had information at the municipal level, with each municipality identified by an official number code. The three datasets were merged using this Brazilian municipal code.

### Variables definition

#### Homicide

A death resulting from injuries inflicted by another person with intent to injure or kill using any means according to the International Classification of Diseases, 10th revision [24], codes X85-Y09. The outcome variable, homicide rate was calculated at the municipality level and directly standardized by age (five year groups) using the WHO population as reference. Age-standardized homicide rate was calculated overall, by sex and age strata (children and adolescents from 0 to 14 years old, young 15–29, adults 30–59, and elderly 60 or over) for each municipality and year of analysis.

#### Hospitalization resulting from violence

Number of hospitalizations using the codes X85-Y09, divided by the number of inhabitants in the municipality per 100,000 inhabitants.

**BFP coverage** was classified, as in previous studies [16,17], into two types of coverage both transformed into percentages:

1. **BFP coverage in the target population** or the number of people receiving the BFP by the total number of eligible people, and
2. **Percentage of municipality inhabitants receiving the BFP benefit** (proxy for level of poverty) or the number of people receiving the benefit by the total population in the municipality, measuring level of socioeconomic deprivation in those areas.

We have also tested two other measures to check if there was a dose-response effect.

**BFP coverage in the target population by varied levels,** 1 = coverage <30%, 2 = coverage between 30–70%, 3 = coverage of over 70%.

**BFP coverage in the target population by length of time,** coverage of over 70% during 0–5 years. Measured from 2006 to 2012.

Other variables that were included in the model as control variables are: the monthly per capita income, measured per each 100 BR$; percentage of unemployed people aged 16 years or over, policing rate (number of police officers in the municipality divided by the population per 100,000 inhabitants); a proxy of guns availability estimated by the percentage of suicide committed with guns among the total suicides in the municipality [25]; percentage of people with low education level estimated as the proportion of individuals 10 years or older with up to eight years of education (nine years is the minimum education required in Brazil); and the urbanization rate, measured as the percentage of individuals living in urban areas. These variables were selected because of previous evidence of an association with homicide [2, 3, 6, 7, 8].

### Statistical analyses

Negative binomial regression models were used to evaluate the effect of these variables on homicide rates. A variable indicating calendar year time-specific effect was introduced into the models to control for the national-level policy changes or secular trends common to all the municipalities [26].

The regression model we applied was: In(Y_it_) = α_i_ + β1BFP_it_ + βnXn_it_ + γ_t_ + u_it_,where Y_it_ is the homicide rate for municipality i in year t, α_i_ is the fixed effect for the municipality i that captured all unobserved time-invariant factors, BFP_it_ was the Bolsa Familia coverage for the municipality i in the year t, Xn_it_ is the value of each n covariate of the model, including all socioeconomic determinants, in the municipality i in the year t, γt was the time-specific effect, and u_it_ was the error.

The adequacy of the specification was tested using the Hausman test and the robustness of the results by difference-in-difference models and other sensitivity analyses. Models with diverse specifications were fitted, including Poisson regressions with robust standard errors. However, we could not adopt the Poisson model because the assumption that the mean is equal to the variance did not hold true in our dataset. In addition, the overdispersion of our dataset suggests Poisson models are not the best or most appropriate methods to use with this datatsets. We have also tested both, negative binomial regression and Poisson, using the Akaike’s Information Criterion (AIC) and the Bayesian Information Criterion (BIC) tests. These tests also confirmed that that the negative binomial regression models best fitted our data [27]. All statistical analyses were conducted using Stata (v.14).

The ethics committee from the London School of Hygiene and Tropical Medicine (LSHTM) approved this study, ethics reference number 11581.

## Results

Table 1 presents homicide and hospitalization from violence rates, proportion of the target population receiving the BFP benefit, and general information on potential controls. Homicide rates increased by 24% overall from 2004 to 2012, BFP coverage in the target population by 47%, and percentage of municipality inhabitants receiving the BFP benefit by 67%. There were improvements in all socioeconomic indicators overall; per capita income increased by 29.4%, unemployment decreased by 36% and percentage of people with low education level by 22% in our period of analyses. Policing rate and guns availability also decreased, by 26.5% and 22.5%, respectively.

**Table 1:** Mean values and SD of selected variables for the Brazilian municipalities (n. 5.507).

Outcomes	2004		2012		Percentage of Change
	Mean	(SD)	Mean	(SD)	
Homicide rate*	14.45	0.25	17.93	0.28	24.03
Homicide rate among men*	25.47	0.46	31.80	0.51	24.87
Homicide rate among women*	3.23	0.14	3.98	0.15	23.32
Hospitalization from violence	5.48	44.29	8.71	85.67	58.92
**Main factors being evaluated**					
BFP coverage of the target population	62.16	0.30	91.15	0.22	46.64
% of inhabitants receiving BFP benefit	20.84	0.19	34.82	0.29	67.07
**Other associated factors**					
Per capita income BR$ (monthly)	395.11	2.83	511.41	3.39	29.43
% unemployed people	8.74	0.06	5.57	0.05	-36.27
% of people with low education level	75.63	0.11	59.12	0.14	-21.83
Policing rate	131.46	2.75	96.66	2.78	-26.47
Guns availability	6.73	0.29	5.21	0.25	-22.50
Urbanization rate	60.89	0.30	65.17	0.29	7.02

Abbreviations: SD = Standard Deviation

*Age standardized rate.

Homicide rates and hospitalization from violence decreased as BFP coverage increased in the target population. An increased percentage of inhabitants receiving the BFP benefit in the municipalities (proxy for level of poverty) was associated with higher homicide rates. We found similar results controlling for both variables in the same model (Table 2) and when the models were stratified by areas with diverse municipal population sizes. Also testing for the municipal proportion of people eligible to participate in the programme.

**Table 2:** Fixed effect regression models for adjusted associations between homicide rates or hospitalizations from violence and BFP coverage and percentage of municipality inhabitants receiving BF in the Brazilian municipalities (as continuous variables), also stratified by municipalities of different population sizes, 2004–2012.

Variable	Model 1		Model 2		Model 3		Population< = 10.000)		Population 10.001–50.000		Population>50.000	
	RR	(95% CI)	RR	(95% CI)	RR	(95% CI)	RR	(95% CI)	RR	(95% CI)	RR	(95% CI)
**Homicide rates**												
BFP coverage of the target population	0.998	(0.998–0.999)	-	-	0.997	(0.996–0.997)	0.997	(0.995–0.999)	0.998	(0.998–0.999)	0.997	(0.996–0.997)
% of inhabitants receiving BFP benefit	-	-	1.005	(1.004–1.006)	1.009	(1.008–1.011)	1.009	(1.006–1.013)	1.010	(1.008–1.012)	1.011	(1.008–1.014)
Per capita income BR$ (monthly)	0.915	(0.902–0.928)	0.927	(0.913–0.940)	0.940	(0.926–0.954)	1.018	(0.963–1.076)	1.001	(1.001–1.002)	0.942	(0.923–0.962)
% unemployed people	0.987	(0.982–0.992)	0.985	(0.980–0.990)	0.985	(0.980–0.989)	0.987	(0.975–1.000)	0.997	(0.989–1.005)	0.996	(0.986–1.006)
Policing rate	1.000	(1.000–1.000)	1.000	(1.000–1.000)	1.000	(1.000–1.000)	1.000	(0.999–1.000)	1.000	(1.000–1.000)	1.000	(1.000–1.000)
Guns availability	1.001	(1.000–1.001)	1.001	(1.000–1.001)	1.001	(1.000–1.001)	1.002	(1.001–1.003)	1.000	(1.000–1.001)	1.001	(1.000–1.001)
% of people with low education level	1.045	(1.041–1.049)	1.040	(1.035–1.044)	1.041	(1.037–1.045)	1.006	(0.992–1.020)	1.010	(1.003–1.017)	1.033	(1.025–1.040)
Urbanization rate	0.995	(0.993–0.997)	0.994	(0.992–0.996)	0.995	(0.992–0.997)	1.010	(1.004–1.016)	1.003	(1.000–1.006)	0.999	(0.994–1.003)
Time (year)	1.122	(1.114–1.130)	1.096	(1.087–1.104)	1.098	(1.090–1.107)	1.007	(0.983–1.032)	1.024	(1.011–1.037)	1.082	(1.064–1.100)
Number of observations	47448		47448		47448		20735		21285		5270	
Number of municipalities	5272		5272		5272		2430		2528		618	
**Hospitalizations from violence**												
BFP coverage of the target population	0.997	(0.996–0.997)	-	-	0.996	(0.995–0.996)	0.997	(0.995–0.998)	0.998	(0.997–0.999)	0.995	(0.993–0.996)
% of inhabitants receiving BFP benefit	-	-	0.999	(0.997–1.000)	1.004	(1.003–1.006)	1.013	(1.009–1.017)	1.006	(1.003–1.008)	1.007	(1.003–1.012)
Per capita income BR$ (monthly)	0.987	(0.971–1.000)	0.983	(0.967–0.999)	1.000	(.984–1.017)	1.061	(1.002–1.123)	1.001	(1.000–1.001)	1.016	(0.989–1.045)
% unemployed people	0.955	(0.950–0.961)	0.953	(0.947–0.958)	0.953	(0.948–0.959)	0.980	(0.968–0.993)	0.999	(0.990–1.008)	0.937	(0.924–0.950)
Policing rate	0.999	(0.999–0.999)	0.999	(0.999–0.999)	0.999	(0.999–0.999)	1.000	(1.000–1.001)	1.000	(1.000–1.000)	1.000	(0.999–1.000)
Guns availability	1.000	(0.999–1.000)	1.000	(0.999–1.000)	1.000	(0.999–1.000)	1.000	(0.999–1.002)	1.000	(0.999–1.001)	1.001	(1.000–1.003)
% of people with low education level	1.048	(1.043–1.052)	1.046	(1.041–1.051)	1.046	(1.041–1.050)	0.976	(0.964–0.988)	0.996	(0.989–1.003)	1.032	(1.022–1.042)
Urbanization rate	0.998	(0.997–1.000)	0.998	(0.996–0.999)	0.999	(0.997–1.000)	0.998	(0.994–1.002)	1.001	(0.999–1.004)	1.000	(0.995–1.006)
Time (year)	1.083	(1.074–1.092)	1.072	(1.062–1.082)	1.072	(1.063–1.082)	0.952	(0.932–0.972)	0.997	(0.983–1.011)	1.016	(0.993–1.040)
Number of observations	45324		45324		45324		19432		20335		5261	
Number of municipalities	5036		5036		5036		2267		2403		617	

Abbreviations: CI = Confidence Interval; RR = Rate Ratio; H = Hospitalization

Model 1: Including only target population coverage; Model 2: including only % of people receiving BF; Model 3: including both.

Increased income and urbanization were associated with decreased homicide rates, while increased gun availability and low levels of schooling increased homicide rates. Policing rates did not have an effect on homicide rates. However, it appears to decrease hospitalizations from violence. Repeating the same analyses separately for municipalities according to population size showed that the magnitude of the associations between homicide and hospitalization rates and BFP coverage remained unaltered, regardless of the size of municipalities. Urbanization rates were associated with increased homicide rates exclusively in the smaller municipalities and unem ployment was not associated with homicide after stratifying (Table 2).

The association between coverage level BFP and homicide rates seemed to follow a dose-response pattern. BFP coverage between 30–70% decreased homicide rates by 16% and hospitalizations from violence by 10%, while coverage of over 70% decreased both by 23% (Table 3). A sensitive analyses, including only municipalities considered in previous study to have accurate data [28] yield similar results (See S1 Table); models for different coverage stratifications (tertiles, quartiles or quintiles) (See S2 Table); and dichotomizing for different cutoffs (See S1 Appendix), also showed that the homicides rates decreased as the BFP coverage in the target population increased.

**Table 3:** Fixed effect regression models for adjusted associations between homicide rates or hospitalizations from violence and BFP coverage in the Brazilian municipalities by coverage level and duration, 2004–2012.

Variable	Model 1				Model 2*			
	Homicide		Hosp. from violence		Homicide		Hosp. from violence	
	RR	(95% CI)	RR	(95% CI)			RR	(95% CI)
1	1.000		1.000		1.000		1.000	
2	0.838	(0.792–0.887)	0.896	(0.828–0.970)	0.834	(0.787–0.883)	0.924	(0.845–1.010)
3	0.772	(0.729–0.818)	0.775	(0.715–0.841)	0.786	(0.743–0.833)	0.864	(0.791–0.943)
4	-	-	-	-	0.769	(0.726–0.814)	0.807	(0.739–0.880)
5	-	-	-	-	0.759	(0.716–0.804)	0.749	(0.685–0.819)
6	-	-	-	-	0.796	(0.750–0.846)	0.777	(0.709–0.851)
% of inhabitants receiving BFP benefit	1.008	(1.007–1.009)	1.003	(1.001–1.004)	1.006	(1.004–1.007)	0.999	(0.997–1.001)
Per capita income BR$	0.937	(0.924–0.951)	0.999	(0.82–1.016)	0.958	(0.939–0.977)	1.003	(0.982–1.025)
% unemployed people	0.985	(0.980–0.990)	0.953	(0.948–0.959)	0.989	(0.982–0.995)	0.947	(0.940–0.954)
Policing rate	1.000	(1.000–1.000)	0.999	(0.999–0.999)	1.000	(1.000–1.000)	0.999	(0.999–0.999)
Guns availability	1.001	(1.000–1.001)	1.000	(0.999–1.000)	1.000	(1.000–1.001)	1.000	(0.999–1.000)
% of people with low education level	1.040	(1.036–1.044)	1.046	(1.041–1.050)	1.034	(1.029–1.039)	1.050	(1.045–1.056)
Urbanization rate	0.994	(0.992–0.996)	0.998	(0.996–1.000)	0.995	(0.992–0.998)	0.998	(0.996–1.000)
Time (year)	1.095	(1.086–1.103)	1.071	(1.061–1.080)	1.094	(1.082–1.107)	1.129	(1.115–1.144)
Number of observations	47488		45324		36351		34244	
Number of municipalities	5227		5036		5193		4892	

Abbreviations: CI = Confidence Interval; RR = Rate Ratio

Model 1: 1 = coverage <30%, 2 = coverage between 30–70%, 3 = coverage of over 70%; Model 2: coverage of over 70% during 0–5 years.

*Model 2 was measured from 2006 to 2012.

The length of time of BFP coverage also influenced the effect for both homicide rates and hospitalizations from violence, with a peak effect at 4 years. Municipalities with one year of coverage at 70% or more had a 17% decrease in homicide rates over the study period while municipalities with two, three, and four years had 21%, 23%, and 24% decreases respectively. Similarly, hospitalizations from violence decreased by: 8% in municipalities with one-year of coverage at 70%; 14% with two-years, 20% with three-years and 25% with 4 years (Table 3). Sensitivity tests using coverages of BPF at 80% and 90% per year yield similar results (See S2 Appendix).

Both male and female homicide rates decreased as BFP coverage in the target population increased; while an increased percentage of municipality inhabitants receiving the BFP benefit was associated with increased male and female homicide rates. Increased unemployment and guns availability appeared to increase female homicide rate while low level of education seemed to increase male homicide rates. Policing rates did not have an effect in any sex (Table 4).

**Table 4:** Fixed effect regression models for adjusted associations between homicide rates and BFP coverage in the Brazilian municipalities by gender, 2004–2012.

Variable	Male analysis model		Female analysis model	
	RR	(95% CI)	RR	(95% CI)
BFP coverage 0–30%	1.000		1.000	
BFP coverage >30–70%	0.837	(0.790–0.888)	0.804	(0.723–0.894)
BFP coverage >70%	0.770	(0.726–0.818)	0.730	(0.655–0.813)
% of inhabitants receiving BFP benefit	1.008	(1.007–1.009)	1.013	(1.010–1.016)
Per capita income BR$ (monthly)	0.938	(0.924–0.952)	1.001	(1.000–1.001)
% unemployed people	0.986	(0.981–0.991)	1.021	(1.008–1.034)
Policing rate	1.000	(1.000–1.000)	1.000	(1.000–1.000)
Guns availability	1.000	(1.000–1.001)	1.003	(1.002–1.004)
% of people with low education level	1.040	(1.035–1.044)	0.997	(0.986–1.007)
Urbanization rate	0.995	(0.992–0.997)	1.001	(0.996–1.007)
Time (year)	1.094	(1.085–1.103)	0.998	(0.976–1.020)
Number of observations	46890		33921	
Number of municipalities	5210		3769	
Number of homicides	338776		35871	

Abbreviations: CI = Confidence Interval; RR = Rate Ratio.

Analyzing by sex and age groupings, among men the effect of BFP coverage was found from 15 years or older and among women from 15 to 59 years old. However, without stratification by sex, BFP of the target population appears to impact on all age groups (See S3 Table and S3 Appendix for discussion). In the difference-in-difference analyses, BFP coverage in the target population was also associated with decreased homicide rates (See S4 Appendix).

## Discussion

Our results show that increases in BFP coverage in the target population is associated with decreased homicide rates and hospitalizations resulting from violence, even after adjusting for other well-known factors associated with homicide. It is interesting to note that the observed effect remains after stratifying by municipal population size, as well as by sex and age groups. This association increases by BFP coverage level and the duration of this coverage. Municipalities with higher BFP coverage had lower homicide rates, longer and higher levels of BFP coverage were associated with a stronger association.

As shown by the Rate Ratios of Table 3, we found an increased effect according to coverage level; in other words, a higher BFP coverage was associated with a decreased homicide rate. For instance, a 16% reduction of homicides rates at BFP coverage levels between 30–70% and a 23% reduction when the BFP coverage was over 70%. Incidentally, the length of time with a high coverage also seems to impact on homicide rates, 21% reduction after 2 years at 70% coverage or over, 23% reduction after 3 years and 24% reduction after 4 years.

The evidence showing an effect of cash transfer programmes, or other similar interventions aimed to improve income, on violence is inconsistent so far. In Chicago, vouchers schemes reduced by 20% violent crimes and arrests [29], in Bogota days after payment had less criminal activities [30]; in the US, decreased earnings among unskilled workers were associated with increased crime [31], and investments in anti-poverty programs were considered as powerful decreasing violence in Guatemala [32]. However, studies in Colombia [30] and Argentina [33] found no effect of cash transfer schemes on homicide rates but a negative association with property crimes. The authors considered that the amount of money on offer was too small to prevent homicides [33].

In Brazil, a high percentage of homicides are related to property crimes and illicit drugs traffiking [34] and BFP was shown to reduce thefts, robberies, vandalism, violent crimes, and drug related offenses [15]. There was previous evidence that BFP reduced murder (homicide with an intention) rates [20] but had no effect on homicide rates when comparisons were made at state level [19]. However, using the 27 Brazilian states as units of analysis might have not provided enough statistical power to detect significant effects at this level and to detect within state variations. This study did not undertake further sensitivity analyses to confirm the results or adjust for important confounders such as sex and age [19, 20], both important factors associated with homicide.

**Fig 1:**
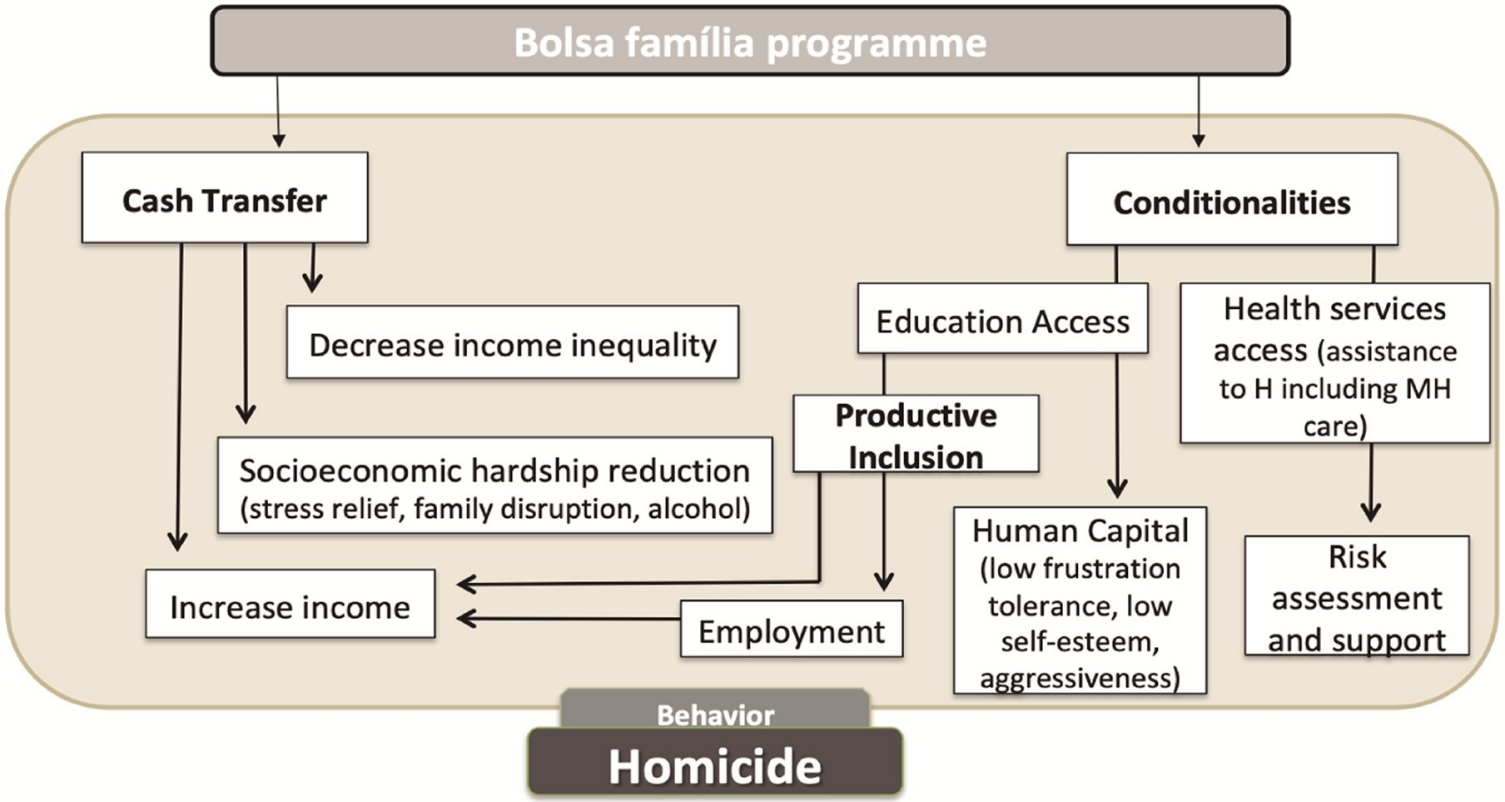
Mechanisms linking the *Bolsa Família Programme* to homicide. H: Health; MH: Mental Health.

BFP could influence homicides through decreasing income inequalities or reducing socioeconomic hardship through increased family income, which might result in stress reduction, less family disruption, or decreased alcohol consumption [35]. Poverty and income inequalities have been associated with violent crimes [21]. BFP can also influence homicide rates indirectly through accomplishing the conditionalities included in the programme. For instance, increasing school attendance and consequently improving educational levels which on its turn can also lead to improving the quality of social networks i.e. making friends at school rather than on the streets [15] and reducing opportunities for certain types of crime and risky behavior, as stated in the “time incapacitation effect” [15]. Participating of the BFP might also decrease violent acts through promoting “productive inclusion” that increases the chances of the beneficiaries to get better jobs and possibly higher incomes and increasing human capital (giving people the ability of reason about getting involved or committing violent acts). In addition, the health conditionality can increase access to health and social care for instance when there is intimate partner violence (IPV). Access to health and social care can increase assessments and/or support for IPV victims, and those seems to be important to prevent female homicide [36]. Fig 1 summarizes some important factors that might be important to prevent homicide.

Among other factors we included in our analytical models as controlling factors, we found that the percentage of municipality inhabitants receiving BFP benefits were positively associated with municipal homicide rates. We used this variable because it is probably the most reliable measure of poverty available and the only one available at the municipal level. In addition, when using an interpolated measure of “percentage eligible” to receive the BFP benefit as exposure of interest, the results remained similar. We also found that low levels of schooling were positively associated with homicide rates and hospitalization resulting from violence. As we mentioned above, increasing the time spent in educational activities might decrease the opportunity for criminal activities and violence [37]. Through the educational conditionality, BFP aims to increase school enrollment and reduce dropout rates [18].

As expected, gun availability was also positively associated with homicide. The implementation of gun control laws in Brazil in 2003 resulted in a reduction of gun-related deaths [38] but gun availability still seem to influence homicide rates in the country. Our results suggest that municipalities with higher gun availability have higher homicide rates.

As widely mentioned in previous literature, we also found that higher income seems to act as a protecting effect on homicide rates. This finding is in keeping with the hypothesis that increasing income through conditional cash transfer programs can be a way to prevent violent deaths.

Urbanization was also associated with higher homicide rates but exclusively among the smaller Brazilian municipalities. It is possible that this effect resulted from the more recent urbanization process taking place in these cities. The more disorganized urbanization process in megacities in Brazil happened before our period of analyses.

Unemployment was found to be negatively associated with homicide rates. However, we urge caution on this finding as this is a complex variable in Brazil where there are large numbers of informal jobs.

### Limitations and strengths of the study

The main limitation of this study is that it is a contextual not an individual level study and therefore no conclusions can be applied to individuals but they relate to municipalities. However, this allowed a country-wide analysis with a higher external validity than classic individual-level studies and an overall understanding of the influences of the BFP at the community level, including spill-over effects. Using the ecological level allowed us to investigate 9 years of longitudinal data, while other designs would have been too costly. Also, studying smaller areas than previous studies had done reduced the possibility of ecological fallacy [39, 40].

The use of mixed ecological designs allowed us to test if BFP coverage would be associated with decreased homicide rates and violence-related hospitalizations over time. We had repeated information from each municipality over time and therefore, this study design allowed—with the use of FE specification along the longitudinality of the panel data—to suggest besides the statistical associations also a possible causal relation between the increase of BFP coverage and the reduction of homicides.

Selection bias could be another limitation. However, the use of fixed effects models allows to some degree controlling for a possible selection bias due to a selective policy implementation, because the fixed effect term of the equation represents these unobserved time-invariant characteristics of the panel [41].

In impact evaluations, fixed effects (FE) models are usually preferred because they permit correlations between the unobserved time-invariant term and the explanatory variables41. In our case, the time-invariant term could represent unobserved characteristics of the municipality such as geographical, historical, socio-cultural or socio-economic characteristics that did not change during the period of the study. In fixed effects models, but not in random ones, those characteristics could be correlated with the treatment variables, such as the BFP. For example, the implementation of the BFP in some municipalities might have influenced certain cultural and geographic factors, which impacted on the development of favelas and these changes determined higher homicides rates. It was not possible to include any of these variables or proxy-variables capable of representing these changes in the model, but we assume a reasonable time-invariance of these factors along the period of the study while the other time-varying socioeconomic factors related to homicides have been included as confounding variables in the model. The FE specification reduces the impact on estimates of omitted variables biases.

This study design and model specifications have been widely used to demonstrate the association of Bolsa Familia and other public health or social interventions on the improvement of several health outcomes in Brazil [16, 17, 42, 43]. For instance, in a study it was used to show the effects of BFP on the reduction of child morbidity and mortality [16], and more recently the effects BFP on Tuberculosis and Leprosy [17], besides its use to demonstrate the effects of other interventions–such as Primary care—in Brazil [44].

Another limitation is that large municipalities and small municipalities have equal weight in the analysis. However, we wanted each municipality to have equivalent importance in the effect assessment of the implementation of BFP. We performed extra analyses grouping municipalities by size to test if our results were influenced by this factor and the results did not appear to differ.

The completeness and quality of the data could also be a potential limitation. However, almost 80% of the Brazilian population live in areas with satisfactory levels of death information [45] and the data of BFP coverage, socioeconomic determinants from the national census, and hospitalizations from Ministry of Health, have all been assessed as having high standards [4, 22]. Moreover, the sensitivity analysis only with municipalities with adequate info had similar results.

One of the main strengths of the current study is the use of panel data analysis rather than traditional cross-sectional data analysis. The use of longitudinal data allowed us to explore the influence of the BFP and social contextual features over time, providing stronger evidence for the association of BFP coverage with homicide rates in Brazil. The use of difference-in-difference to test the association resulting in similar results provides additional strength to the findings.

## Conclusion

At the municipal level, take up of the Brazilian conditional cash transfer programme seems associated to a decrease in homicide rates and in hospitalizations from violence, in both sexes, and across all age groups. Assuming this effect to be causal, the programme would have prevented approximately 58 460 deaths from 2004–2012.

Social protection interventions, which reduce poverty and socioeconomic inequalities, can contribute to decreasing mortality from homicides and hospitalization related to violence in Brazil and possibly in other low-middle-income-countries with similar programs.

## Supporting information

S1 Table Fixed effect regression models for adjusted associations between homicide rates and BFP coverage including only municipalities with accurate vital information in Brazil, 2004–2012. (DOCX)

S2 Table Fixed effect regression models for adjusted associations between homicide rates or hospitalizations from violence and BFP coverage in the Brazilian municipalities by coverage level and duration, 2004–2012. (DOCX)

S3 Table Fixed effect regression models for adjusted associations between homicide rates and BFP coverage in the Brazilian municipalities overall and by sex and age, 2004–2012. (DOCX)

S1 Appendix Sensitivity analysis dichotomizing for different cutoffs of BFP coverage. (DOCX)

S2 Appendix Sensitivity tests using coverages of BPF at 80% and 90% per year. (DOCX)

S3 Appendix Discussion for the analyses by sex and age discussion. (DOCX)

S4 Appendix Difference-in-difference analyses for the BFP coverage in the target population. (DOCX)
