# Supplementary material for: Conditional cash transfer programme: Impact on homicide rates and hospitalisations from violence in Brazil
Source: PLoS One. 2018 Dec 31;13(12):e0208925. doi: 10.1371/journal.pone.0208925 (PMC6312285; doi:10.1371/journal.pone.0208925)
Supplement: S1 Table — (DOCX) [file pone.0208925.s001.docx]

**S1 Table**. Fixed effect regression models for adjusted associations between homicide rates and BFP coverage including only municipalities with accurate vital information in Brazil, 2004-2012.

| Variable | RR | (95% CI) |
| --- | --- | --- |
| BFP coverage 0-30% | 1.000 |  |
| BFP coverage >30-70% | 0.875 | (0.805-0.951) |
| BFP coverage >70% | 0.815 | (0.746-0.891) |
| % of municipality inhabitants receiving BF | 1.009 | (1.006-1.012) |
| Per capita income BR$ | 0.998 | (0.998-0.998) |
| % unemployed people | 0.973 | (0.959-0.987) |
| Policing rate | 1.000 | (1.000-1.000) |
| Guns availability | 1.001 | (1.000-1.001) |
| % of people with low education level | 1.037 | (1.026-1.048) |
| Urbanization rate | 0.983 | (0.975-0.991) |
| Time (year) | 1.086 | (1.065-1.109) |
|  |  |  |
| Number of observations | 11025 |  |
| Number of municipalities | 1575 |  |
| Abbreviations: CI = Confidence Interval; RR = Rate Ratio | | |
