# Supplementary material for: Conditional cash transfer programme: Impact on homicide rates and hospitalisations from violence in Brazil
Source: PLoS One. 2018 Dec 31;13(12):e0208925. doi: 10.1371/journal.pone.0208925 (PMC6312285; doi:10.1371/journal.pone.0208925)
Supplement: S2 Table — (DOCX) [file pone.0208925.s002.docx]

**S2 Table.** Fixed effect regression models for adjusted associations between homicide rates or hospitalizations from violence and BFP coverage in the Brazilian municipalities by coverage level and duration, 2004-2012.

|  | | | | | | | | |
| --- | --- | --- | --- | --- | --- | --- | --- | --- |
|  | Tercile | |  | Quartile | |  | Quintile | |
| Variable |  |  |  | RR | (95% CI) |  | RR | (95% CI) |
| **Homicide rates** |  |  |  |  |  |  |  |  |
| 1 | 1.000 |  |  | 1.000 |  |  | 1.000 |  |
| 2 | 0.955 | (0.924-0.955) |  | 0.937 | (0.902-0.937) |  | 0.938 | (0.919-0.957) |
| 3 | 0.975 | (0.936-0.975) |  | 0.931 | (0.892-0.931) |  | 0.912 | (0.892-0.933) |
| 4 | - | - |  | 0.933 | (0.888-0.933) |  | 0.910 | (0.888-0.933) |
| 5 | - | - |  | - | - |  | 0.904 | (0.880-0.929) |
| % of municipality inhabitants receiving BF | 1.007 | (1.005-1.007) |  | 1.009 | (1.006-1.009) |  | 1.007 | (1.006-1.009) |
| Per capita income BR$ | 0.999 | (0.999-0.999) |  | 0.999 | (0.999-0.999) |  | 0.999 | (0.999-0.999) |
| % unemployed people | 0.989 | (0.980-0.989) |  | 0.990 | (0.980-0.990) |  | 0.985 | (0.980-0.990) |
| Policing rate | 1.000 | (1.000-1.000) |  | 1.000 | (1.000-1.000) |  | 1.000 | (1.000-1.000) |
| Guns availability | 1.001 | (1.000-1.001) |  | 1.001 | (1.000-1.001) |  | 1.001 | (1.000-1.001) |
| % of people with low education level | 1.045 | (1.037-1.045) |  | 1.045 | (1.037-1.045) |  | 1.041 | (1.037-1.045) |
| Urbanization rate | 0.996 | (0.992-0.996) |  | 0.997 | (0.992-0.997) |  | 0.994 | (0.992-0.997) |
| Time (year) | 1.107 | (1.090-1.107) |  | 1.108 | (1.091-1.108) |  | 1.099 | (1.091-1.108) |
|  |  |  |  |  |  |  |  |  |
| Number of observations | 47448 |  |  | 47448 |  |  | 47448 |  |
| Number of municipalities | 5272 |  |  | 5272 |  |  | 5272 |  |
| Abbreviations: RR = Rate Ratio; CI = Confidence Interval | | |  |  |  |  |  |  |
