# Supplementary material for: Conditional cash transfer programme: Impact on homicide rates and hospitalisations from violence in Brazil
Source: PLoS One. 2018 Dec 31;13(12):e0208925. doi: 10.1371/journal.pone.0208925 (PMC6312285; doi:10.1371/journal.pone.0208925)
Supplement: S3 Table — (DOCX) [file pone.0208925.s003.docx]

**S5 Table.** Fixed effect regression models for adjusted associations between homicide rates and BFP coverage in the Brazilian municipalities overall and by sex and age, 2004-2012.

|  | 0-14 years old | |  | 15-29 years old | |  | 30-44 years old | |  | 45-59 years old | |  | 60 or over | |
| --- | --- | --- | --- | --- | --- | --- | --- | --- | --- | --- | --- | --- | --- | --- |
| Variable | RR | (95% CI) |  | RR | (95% CI) |  | RR | (95% CI) |  | RR | (95% CI) |  | RR | (95% CI) |
| **BFP coverage of the target population** |  |  |  |  |  |  |  |  |  |  |  |  |  |  |
| Male | 0.997 | (0.990-1.005) |  | 0.996 | (0.995-0.997) |  | 0.996 | (0.995-0.996) |  | 0.996 | (0.995-0.997) |  | 0.998 | (0.997-0.999) |
| Female | 0.993 | (0.985-1.002) |  | 0.995 | (0.993-0.996) |  | 0.995 | (0.994-0.997) |  | 0.995 | (0.992-0.998) |  | 1.000 | (0.996-1.004) |
|  |  |  |  |  |  |  |  |  |  |  |  |  |  |  |
| Total | 0.996 | (0.994-0.998) |  | 0.996 | (0.996-0.997) |  | 0.996 | (0.995-0.996) |  | 0.996 | (0.995-0.997) |  | 0.998 | (0.997-0.999) |
| % of municipality inhabitants receiving BF | 1.017 | (1.010-1.024) |  | 1.010 | (1.008-1.012) |  | 1.013 | (1.011-1.015) |  | 1.015 | (1.012-1.018) |  | 1.007 | (1.003-1.012) |
| Per capita income BR$ (monthly) | 1.000 | (0.999-1.001) |  | 0.999 | (0.999-0.999) |  | 0.999 | (0.999-1.000) |  | 1.000 | (0.999-1.000) |  | 0.999 | (0.999-1.000) |
| % unemployed people | 1.008 | (0.981-1.036) |  | 0.979 | (0.972-0.985) |  | 0.997 | (0.989-1.004) |  | 1.022 | (1.010-1.033) |  | 1.014 | (0.997-1.031) |
| Policing rate | 1.000 | (0.999-1.000) |  | 1.000 | (1.000-1.000) |  | 1.000 | (1.000-1.000) |  | 1.000 | (1.000-1.000) |  | 1.000 | (1.000-1.001) |
| Guns availability | 1.001 | (0.999-1.002) |  | 1.001 | (1.000-1.001) |  | 1.001 | (1.000-1.001) |  | 1.000 | (0.999-1.001) |  | 1.001 | (1.000-1.002) |
| % of people with low education level | 0.996 | (0.975-1.018) |  | 1.057 | (1.051-1.062) |  | 1.036 | (1.030-1.042) |  | 1.027 | (1.017-1.037) |  | 1.009 | (0.994-1.024) |
| Urbanization rate | 1.000 | (0.988-1.014) |  | 0.991 | (0.988-0.994) |  | 0.985 | (0.981-0.989) |  | 0.994 | (0.987-1.000) |  | 0.995 | (0.985-1.005) |
| Time (year) | 1.029 | (0.987-1.074) |  | 1.145 | (1.133-1.158) |  | 1.095 | (1.081-1.108) |  | 1.049 | (1.029-1.070) |  | 1.024 | (0.994-1.055) |
|  |  |  |  |  |  |  |  |  |  |  |  |  |  |  |
| Number of observations | 14598 |  |  | 42111 |  |  | 41859 |  |  | 36414 |  |  | 29700 |  |
| Number of municipalities | 1622 |  |  | 4679 |  |  | 4651 |  |  | 4046 |  |  | 3300 |  |
| Number of homicides | 7266 |  |  | 244721 |  |  | 126634 |  |  | 44701 |  |  | 16591 |  |

Abbreviations: RR = Rate Ratio; CI = Confidence Interval

*The controlling variables’ results are showed for the total outcome (female and male together) as well as the number of observations, municipalities and homicides.
