# Supplementary material for: Conditional cash transfer programme: Impact on homicide rates and hospitalisations from violence in Brazil
Source: PLoS One. 2018 Dec 31;13(12):e0208925. doi: 10.1371/journal.pone.0208925 (PMC6312285; doi:10.1371/journal.pone.0208925)
Supplement: S1 Appendix — (DOCX) [file pone.0208925.s004.docx]

**S3 Appendix.** Sensitivity analysis dichotomizing for different cutoffs of BFP coverage

. xtnbreg obi_st_ST BFcat5 cobBFmunlim rendapcapipol desempol txpolic perc_s_arma b_escpol urbapol yearnew, exposure(pop_T) fe ir

note: you are responsible for interpretation of non-count dep. variable

note: 235 groups (2115 obs) dropped because of all zero outcomes

Conditional FE negative binomial regression Number of obs = 47,448

Group variable: cd Number of groups = 5,272

Obs per group:

min = 9

avg = 9.0

max = 9

Wald chi2(9) = 2493.94

Log likelihood = -69210.892 Prob > chi2 = 0.0000

-------------------------------------------------------------------------------

obi_st_ST | IRR Std. Err. z P>|z| [95% Conf. Interval]

--------------+----------------------------------------------------------------

BFcat5 | .8390644 .0134367 -10.96 0.000 .813138 .8658174

cobBFmunlim | 1.007263 .0006058 12.03 0.000 1.006077 1.008451

rendapcapipol | .9993519 .0000752 -8.61 0.000 .9992045 .9994994

desempol | .9843471 .0025058 -6.20 0.000 .9794481 .9892707

txpolic | .9999066 .0000502 -1.86 0.063 .9998082 1.000005

perc_s_arma | 1.000667 .0001651 4.04 0.000 1.000344 1.000991

b_escpol | 1.039054 .0020816 19.12 0.000 1.034982 1.043142

urbapol | .9939464 .0010761 -5.61 0.000 .9918395 .9960577

yearnew | 1.092709 .0042857 22.61 0.000 1.084341 1.101141

_cons | .0000305 6.02e-06 -52.61 0.000 .0000207 .0000449

ln(pop_T) | 1 (exposure)

-------------------------------------------------------------------------------

. xtnbreg obi_st_ST BFcat4 cobBFmunlim rendapcapipol desempol txpolic perc_s_arma b_escpol urbapol yearnew, exposure(pop_T) fe ir

note: you are responsible for interpretation of non-count dep. variable

note: 235 groups (2115 obs) dropped because of all zero outcomes

Conditional FE negative binomial regression Number of obs = 47,448

Group variable: cd Number of groups = 5,272

Obs per group:

min = 9

avg = 9.0

max = 9

Wald chi2(9) = 2479.93

Log likelihood = -69221.661 Prob > chi2 = 0.0000

-------------------------------------------------------------------------------

obi_st_ST | IRR Std. Err. z P>|z| [95% Conf. Interval]

--------------+----------------------------------------------------------------

BFcat4 | .9152113 .0084029 -9.65 0.000 .8988893 .9318297

cobBFmunlim | 1.007237 .0006181 11.75 0.000 1.006027 1.008449

rendapcapipol | .9992955 .0000746 -9.44 0.000 .9991492 .9994418

desempol | .9847442 .0024909 -6.08 0.000 .9798742 .9896383

txpolic | .9999243 .0000502 -1.51 0.132 .9998258 1.000023

perc_s_arma | 1.00067 .0001649 4.06 0.000 1.000347 1.000993

b_escpol | 1.040761 .0020895 19.90 0.000 1.036673 1.044864

urbapol | .9944097 .0010802 -5.16 0.000 .9922949 .9965291

yearnew | 1.098565 .0043163 23.93 0.000 1.090137 1.107057

_cons | .0000241 4.77e-06 -53.69 0.000 .0000163 .0000355

ln(pop_T) | 1 (exposure)

-------------------------------------------------------------------------------

. xtnbreg obi_st_ST BFcat3 cobBFmunlim rendapcapipol desempol txpolic perc_s_arma b_escpol urbapol yearnew, exposure(pop_T) fe ir

note: you are responsible for interpretation of non-count dep. variable

note: 235 groups (2115 obs) dropped because of all zero outcomes

Conditional FE negative binomial regression Number of obs = 47,448

Group variable: cd Number of groups = 5,272

Obs per group:

min = 9

avg = 9.0

max = 9

Wald chi2(9) = 2463.86

Log likelihood = -69229.939 Prob > chi2 = 0.0000

-------------------------------------------------------------------------------

obi_st_ST | IRR Std. Err. z P>|z| [95% Conf. Interval]

--------------+----------------------------------------------------------------

BFcat3 | .9158303 .0091894 -8.76 0.000 .8979955 .9340194

cobBFmunlim | 1.007031 .000618 11.42 0.000 1.005821 1.008243

rendapcapipol | .9993097 .0000752 -9.17 0.000 .9991622 .9994572

desempol | .9849925 .0024999 -5.96 0.000 .980105 .9899044

txpolic | .9999109 .0000502 -1.77 0.076 .9998126 1.000009

perc_s_arma | 1.000668 .000165 4.05 0.000 1.000345 1.000992

b_escpol | 1.040194 .0020849 19.66 0.000 1.036115 1.044288

urbapol | .994102 .0010782 -5.45 0.000 .991991 .9962175

yearnew | 1.096507 .0042951 23.52 0.000 1.088121 1.104957

_cons | .0000258 5.10e-06 -53.42 0.000 .0000175 .000038

ln(pop_T) | 1 (exposure)

-------------------------------------------------------------------------------
