# Supplementary material for: Conditional cash transfer programme: Impact on homicide rates and hospitalisations from violence in Brazil
Source: PLoS One. 2018 Dec 31;13(12):e0208925. doi: 10.1371/journal.pone.0208925 (PMC6312285; doi:10.1371/journal.pone.0208925)
Supplement: S2 Appendix — (DOCX) [file pone.0208925.s005.docx]

**S4 Appendix.** Sensitivity tests using coverages of BPF at 80% and 90% per year

. xi: xtnbreg obi_st_ST i.Bfcatdur_80 cobBFmunlim rendapcapipol desempol txpolic perc_s_arma b_escpol urb

> apol yearnew if year>=2005, exposure(pop_T) fe ir

i.Bfcatdur_80 _IBfcatdur__0-4 (naturally coded; _IBfcatdur__0 omitted)

note: you are responsible for interpretation of non-count dep. variable

note: 269 groups (2152 obs) dropped because of all zero outcomes

Conditional FE negative binomial regression Number of obs = 41,904

Group variable: cd Number of groups = 5,238

Obs per group:

min = 8

avg = 8.0

max = 8

Wald chi2(12) = 1519.09

Log likelihood = -59867.089 Prob > chi2 = 0.0000

-------------------------------------------------------------------------------

obi_st_ST | IRR Std. Err. z P>|z| [95% Conf. Interval]

--------------+----------------------------------------------------------------

_IBfcatdur__1 | .9137405 .0132936 -6.20 0.000 .8880536 .9401704

_IBfcatdur__2 | .875265 .0131022 -8.90 0.000 .8499582 .9013253

_IBfcatdur__3 | .8558401 .0132072 -10.09 0.000 .8303421 .8821212

_IBfcatdur__4 | .9001869 .0150172 -6.30 0.000 .8712298 .9301065

cobBFmunlim | 1.006276 .0006849 9.19 0.000 1.004934 1.007619

rendapcapipol | .9994969 .0000869 -5.79 0.000 .9993265 .9996673

desempol | .9859131 .0028423 -4.92 0.000 .9803579 .9914997

txpolic | .9998115 .0000563 -3.35 0.001 .9997012 .9999217

perc_s_arma | 1.000612 .0001742 3.52 0.000 1.000271 1.000953

b_escpol | 1.035203 .0023089 15.51 0.000 1.030687 1.039738

urbapol | .9947829 .0012092 -4.30 0.000 .9924156 .9971558

yearnew | 1.088489 .005009 18.43 0.000 1.078716 1.098351

_cons | .0000365 8.09e-06 -46.10 0.000 .0000236 .0000563

ln(pop_T) | 1 (exposure)

-------------------------------------------------------------------------------

. xi: xtnbreg obi_st_ST i.Bfcatdur_90 cobBFmunlim rendapcapipol desempol txpolic perc_s_arma b_escpol urb

> apol yearnew if year>=2005, exposure(pop_T) fe ir

i.Bfcatdur_90 _IBfcatdur__0-4 (naturally coded; _IBfcatdur__0 omitted)

note: you are responsible for interpretation of non-count dep. variable

note: 269 groups (2152 obs) dropped because of all zero outcomes

Conditional FE negative binomial regression Number of obs = 41,904

Group variable: cd Number of groups = 5,238

Obs per group:

min = 8

avg = 8.0

max = 8

Wald chi2(12) = 1495.85

Log likelihood = -59891.758 Prob > chi2 = 0.0000

-------------------------------------------------------------------------------

obi_st_ST | IRR Std. Err. z P>|z| [95% Conf. Interval]

--------------+----------------------------------------------------------------

_IBfcatdur__1 | .9735861 .0119958 -2.17 0.030 .9503564 .9973835

_IBfcatdur__2 | .9395932 .0120161 -4.87 0.000 .9163348 .963442

_IBfcatdur__3 | .9288735 .0124818 -5.49 0.000 .9047289 .9536625

_IBfcatdur__4 | .9906999 .0148265 -0.62 0.532 .9620624 1.02019

cobBFmunlim | 1.004899 .00068 7.22 0.000 1.003567 1.006232

rendapcapipol | .9994904 .0000869 -5.87 0.000 .9993202 .9996607

desempol | .9855355 .0028602 -5.02 0.000 .9799455 .9911574

txpolic | .9997817 .0000564 -3.87 0.000 .9996712 .9998921

perc_s_arma | 1.000593 .0001747 3.39 0.001 1.000251 1.000935

b_escpol | 1.035231 .0023179 15.46 0.000 1.030698 1.039784

urbapol | .9945544 .0012069 -4.50 0.000 .9921918 .9969227

yearnew | 1.085743 .0049983 17.87 0.000 1.07599 1.095584

_cons | .0000362 8.05e-06 -45.95 0.000 .0000234 .0000559

ln(pop_T) | 1 (exposure)

-------------------------------------------------------------------------------
