# Supplementary material for: Conditional cash transfer programme: Impact on homicide rates and hospitalisations from violence in Brazil
Source: PLoS One. 2018 Dec 31;13(12):e0208925. doi: 10.1371/journal.pone.0208925 (PMC6312285; doi:10.1371/journal.pone.0208925)
Supplement: S3 Appendix — (DOCX) [file pone.0208925.s006.docx]

**S6 Appendix.** Discussion for the analyses by sex and age discussion

Our results show that BFP coverage decreases both, male and female homicide rates. Among men, the protection effect was found from 15 years or older. However, the limited number of municipalities in the model performed for 0-14 years old may have influenced this result (374 municipalities). Among woman the same reason may explain the lack of effect in the first (0-14 years old) and last age group (60 or more). Analyzing without stratification by sex, BFP of target population has impacted at all age groups.

Male and female homicides generally have diverse causes. While male homicide can be related to criminal activities, most of the violence committed against women in Brazil, including homicides is committed by their partners^1^. Homicide of women is the ultimate consequence of IPV^2^.

In Mexico, the cash transfer reduced IPV by 5-7%^3^ and by 6-7% in Ecuador^4^. In our results BFP in Brazil reduced female homicide rates by 27% at the highest level of coverage (over 70%). Though, in cases where violence is committed by the partner to extract money the introduction of cash through the woman could also result in even more violence. However, overall CCT reduces multiple forms of IPV^4^.

The BFP benefit is mainly paid to the woman and can therefore affect the families’ power dynamics, strengthening female bargaining power and decreasing their partners controlling behavior. These factors have been assumed in the literature as some of the possible reasons why cash transfers decrease IPV^4^. It has been shown that women in beneficiary households are less likely to be victims of physical abuse than non-beneficiaries^3,4,5^.

It has been said that since female homicide commonly represents the result of a long history of abuse, strategies to reduce risk include: increasing investment in IPV prevention which can be achieved by increasing women’s income; increased risk assessment at diverse points of care and support for women experiencing intimate partner violence, among others^2^. The BFPcould then help prevent female homicide by not exclusively increasing income, but also by increasing the beneficiary’s access to risk assessment and support, through the increase of health care assistance.

Violence against women is high related to economic factors. Raising women’s economic self-sufficiency by creating opportunities for them to become financially independent is still the best way of preventing violence against women^6^. Besides the effect similar in both sex, BFP had also similar effect over the range of age groups. This effect is possibly resulted of the capacity of the BFP not exclusively increase income generation, but also increase the human capital at all age groups.

References

1. Waiselfisz, Julio Jacobo. Mapa da Violência 2015: Homicídio de mulheres no Brasil Disponível em [www.mapadaviolencia.org.br](http://www.mapadaviolencia.org.br)
2. Stöckl, Heidi, Karen Devries, Alexandra Rotstein, Naeemah Abrahams, Jacquelyn Campbell, Charlotte Watts, and Claudia Garcia Moreno. 2013. The global prevalence of intimate partner homicide: a systematic review. The Lancet.
3. Hidrobo, Melissa and Lia Fernald (2012). “Cash Transfers and Domestic Violence.” *Journal of Health Economics*, 32(1): 304–319.
4. Hidrobo, Melissa, Amber Peterman, and Lori Heise (2015). “The effect of cash, vouchers and food transfers on intimate partner violence: Evidence from a randomized experiment in Northern Ecuador.” Washington, DC: International Food Policy Research Institute. Unpublished manuscript.
5. Bobonis, Gustavo J., Melissa González-Brenes, and Roberto Castro (2013). “Public Transfers and Domestic Violence: The Roles of Private Information and Spousal Control.” *American Economic Journal: Economic Policy*, 5(1): 179–205.
6. Ogrodnik, Corinne and Borzutzky, Silvia (2011). Women under Attack: Violence and Poverty in Guatemala. Journal of International Women's Studies, 12(1), 55-67. Available at: http://vc.bridgew.edu/jiws/vol12/iss1/4
