# Supplementary material for: Conditional cash transfer programme: Impact on homicide rates and hospitalisations from violence in Brazil
Source: PLoS One. 2018 Dec 31;13(12):e0208925. doi: 10.1371/journal.pone.0208925 (PMC6312285; doi:10.1371/journal.pone.0208925)
Supplement: S4 Appendix — (DOCX) [file pone.0208925.s007.docx]

**S7 Appendix.** Difference-in-difference analyses for the BFP coverage in the target population

reg MRstHOMIC_T_diff bflim_diff cobBFmunlim_diff rendapcapipol_diff desempol_diff txpolic_diff perc_s_arma_diff b_escpol_diff urbapol_diff

Source | SS df MS Number of obs = 5,507

-------------+---------------------------------- F(8, 5498) = 13.29

Model | 55863.2456 8 6982.9057 Prob > F = 0.0000

Residual | 2888844.86 5,498 525.435587 R-squared = 0.0190

-------------+---------------------------------- Adj R-squared = 0.0175

Total | 2944708.11 5,506 534.818036 Root MSE = 22.922

------------------------------------------------------------------------------------

MRstHOMIC_T_diff | Coef. Std. Err. t P>|t| [95% Conf. Interval]

-------------------+----------------------------------------------------------------

bflim_diff | -.0559602 .0149744 -3.74 0.000 -.0853159 -.0266044

cobBFmunlim_diff | .2416391 .0328278 7.36 0.000 .1772836 .3059946

rendapcapipol_diff | -.0045074 .0039487 -1.14 0.254 -.0122485 .0032336

desempol_diff | -.4665725 .0810646 -5.76 0.000 -.6254913 -.3076537

txpolic_diff | .0001289 .0027281 0.05 0.962 -.0052193 .0054771

perc_s_arma_diff | .0130502 .0113409 1.15 0.250 -.0091825 .0352829

b_escpol_diff | .1879078 .0923802 2.03 0.042 .0068061 .3690095

urbapol_diff | -.0113855 .0617729 -0.18 0.854 -.1324849 .1097139

_cons | 3.937232 1.691339 2.33 0.020 .6215386 7.252925

------------------------------------------------------------------------------------
